# Obstetrics: A Text-Book for the Use of Students and Practitioners

**Published:** 1903-12

**Authors:** 


**Obstetrics: A Text-book for the use of Students and Practitioners.** By J. WHITRIDGE WILLIAMS. Pp. xxii., 845. New York and London. D. Appleton & Co. 1903.

This is a large volume. It is written in the American discursive style, in excellent English, and without the objectionable phonetic spelling.

The illustrations are excellent, artistic, and in great abundance.

The chief fault in the book lies in the deficiency of detail in treatment. Often after a masterly article on a particular subject, treating the history, pathology, course, complications, &c., the treatment is only indicated on general principles, which would be of very little practical value to a practitioner. For instance, there is no mention of the difficulty experienced in getting rid of the whole mass in hydatidiform mole. The treatment of abortion is discussed in all its varieties in a little over a page.

The chapter on microscopical anatomy is particularly good, a subject often too scantily dealt with in text-books. Whether advisedly or not, the anatomy of the pelvic organs has little said about it.

The main part of the book is pleasant reading, exceedingly clear, and without many marked differences of opinion from most writers.

The binder is considered inadvisable before the tenth day. Ergot is never to be used before the third stage, and even then only if there are special indications.

The care of the puerperium, a most important chapter, is too short and lacking in detail.

There is a long and interesting chapter on forceps, but the indications for their application are hard to find. Extra-uterine gestation is very fully discussed, and the importance of immediate operation on unruptured cases insisted on.

The author is very strongly opposed to symphysiotomy, ending the chapter with these words: "Personally I do not expect to perform symphysiotomy under any circumstances, and consider that the present enthusiasm for it will eventually disappear."

After a careful analysis of the theories of eclampsia, he decides against the kidneys as the probable starting-point of the disease, and thinks that one day will prove it to be an auto-intoxication by materials normally got rid of, and which, being retained, give rise to the lesions found in the kidneys, liver, and brain.

The author does not give a clear account of the premonitory symptoms of this disease, and says almost nothing of the importance of watching the diminution of urea as an indication for the induction of labour.

Placenta prævia when diagnosed indicates the necessity for immediate termination of pregnancy, packing being but seldom advisable.

He is strongly averse to intrauterine douches with antiseptic lotions, considering that they are often dangerous and not so efficacious as the use of the sterile finger and boiled water.

Puerperal insanity is dismissed in a few words.

In spite of the above criticisms, the book is one of the most readable, interesting, and useful which we have at present, and although it may be somewhat too advanced for the student, it cannot fail to become popular with the practitioner.

